# Strong antibiotic production is correlated with highly active oxidative metabolism in *Streptomyces coelicolor* M145

**DOI:** 10.1038/s41598-017-00259-9

**Published:** 2017-03-15

**Authors:** Catherine Esnault, Thierry Dulermo, Aleksey Smirnov, Ahmed Askora, Michelle David, Ariane Deniset-Besseau, Ian-Barry Holland, Marie-Joelle Virolle

**Affiliations:** 1 Group “Energetic Metabolism of Streptomyces”, Institute for Integrative Biology of the Cell (I2BC), CEA, CNRS, Univ. Paris-Sud, INRA, Université Paris-Saclay, F-91198 Gif-sur-Yvette Cedex, France; 2 Department of Microbiology, Faculty of Science, Zagazig University, Zagazig, 44519 Egypt; 3 Laboratoire de Chimie Physique, Université Paris-Sud, F-91405 Orsay Cedex, France; 4 Sorbonne Universités, UPMC Univ Paris 06, UFR 927, Sciences de la vie, F-75005 Paris, France

## Abstract

The *Streptomyces* genus is well known for its ability to produce bio-active secondary metabolites of great medical interest. However, the metabolic features accompanying these bio-productions remain to be defined. In this study, the comparison of related model strains producing differing levels of actinorhoddin (ACT), showed that *S. lividans*, a weak producer, had high TriAcylGlycerol (TAG) content indicative of a glycolytic metabolism. In contrast, the strong producer, *S. coelicolor*, was characterized by low TAG content, active consumption of its polyphosphate (PolyP) stores and extremely high ATP/ADP ratios. This indicated highly active oxidative metabolism that was correlated with induction of ACT biosynthesis. Interestingly, in conditions of phosphate limitation, the *ppk* mutant had TAG content and ACT production levels intermediary between those of *S. lividans* and *S. coelicolor*. This strain was characterized by high ADP levels indicating that Ppk was acting as an Adenosine Di Phosphate Kinase. Its absence resulted in energetic stress that is proposed to trigger an activation of oxidative metabolism to restore its energetic balance. This process, which is correlated with ACT biosynthesis, requires acetylCoA to fuel the Krebs cycle and phosphate for ATP generation by the ATP synthase coupled to the respiratory chain, resulting in low TAG and polyP content of the ACT producing strains.

## Introduction

Novel antibiotics are urgently needed to face the threat posed by multi-resistant pathogenic bacteria to human health. The filamentous Gram + soil bacteria, *Streptomyces*, already provides more than 60% of the antibiotics used in modern medicine^1–3^. Although each *Streptomyces* species is known to produce 2 to 4 different bio-active molecules, the analysis of the sequence of the genomes of several members of this genus reveals that each encodes 20 to 40 biosynthetic pathways directing the synthesis of potentially useful secondary metabolites^4–7^. This indicates that only 10% of the huge metabolic diversity of this genera is known and thus exploited. In fact, most of the biosynthetic pathways detected *in silico* are poorly expressed in the usual laboratory conditions. In order to access this huge metabolic diversity and discover much needed novel antibiotics, innovative strategies must be found to enhance their production^8–10^. Even if significant progress has been made in the analysis of complex growth and development of these bacteria^11^, a better understanding of the links between primary and secondary metabolism and the characterization of specific metabolic features of strong antibiotic producers is necessary in order to identify the genetic basis and environmental conditions that govern this ability.

*S. coelicolor* M145^12^ and its close relative *S. lividans* TK24^13^ are the model strains extensively used in the field to address these questions. Indeed both strains possess the same pathways for the biosynthesis of well-characterized secondary metabolites of the polyketide (ACT) or peptidyl (CDA, RED) families but the expression of these pathways is high in *S. coelicolor* and relatively low in *S. lividans*. Interestingly, disruption of the *ppk* gene of *S. lividans* resulted in a *S. coelicolo* ***r*** **-**like phenotype regarding its ability to produce antibiotics^14^. The *ppk* gene belongs to the *pho* regulon and is thus mainly expressed under Pi limitation^15, 16^, a condition known to correlate with energetic stress. This gene encodes an enzyme with two distinct activities *in vitro*. Ppk acts as a polyphosphate kinase (PPK), polymerizing the γ phosphate of ATP to yield polyphosphate and ADP when the ATP/ADP ratio in the reaction mix is high. On the other hand, when this ratio is low, the enzyme acts as an Adenosine DiPhosphate Kinase (ADPK) regenerating ATP from ADP and polyphosphate^14^. However, the *in vivo* function of this enzyme remained a pending question that was addressed in this issue. Furthermore, we established previously that the two antibiotic producing strains, *S. coelicolor* and the *ppk* mutant of *S. lividans*, had a lower content of storage lipids of the TriAcylGlycerol (TAG) family than the non-producer, *S. lividans* WT^17^. The content of storage lipids is thus negatively correlated with the production of secondary metabolites. A putative link between storage lipid metabolism and antibiotic production was already anticipated 20 years ago by Olukoshi and Packter^18, 19^ and impaired biosynthesis^20, 21^ or enhanced degradation^22^ of fatty acids was shown to be correlated with increased and reduced antibiotic biosynthesis, respectively.

In this study, in order to gain a better understanding of what governs antibiotic production, we sought to establish the similar and the different features of carbon, phosphate and energetic metabolism of the weak, medium and strong antibiotic producing strains, *S. lividans* TK24 WT, its *ppk* mutant and *S. coelicolor* M145, respectively. Strains were grown in media containing limited or proficient amount of phosphate, conditions known to promote and repress antibiotic production, respectively^23–25^. Pi consumption, as well as intracellular concentrations of free phosphate, polyP, ATP and ADP were measured in these two conditions. Glucose consumption, total lipid content, as well as antibiotic production were also assayed at discrete points throughout growth in the same conditions. These comparative studies indicated that TAG accumulation is supported by a glycolytic metabolism whereas antibiotic production is clearly associated with a highly active oxidative metabolism. In addition, our results demonstrated that Ppk acts as an ADPK *in vivo*, confirming that energetic stress is an important trigger an antibiotic production. The function of the secondary metabolites produced in this context for the producer itself is discussed.

## Results

### TAG accumulation is negatively correlated with antibiotic production

**Figure 1:**
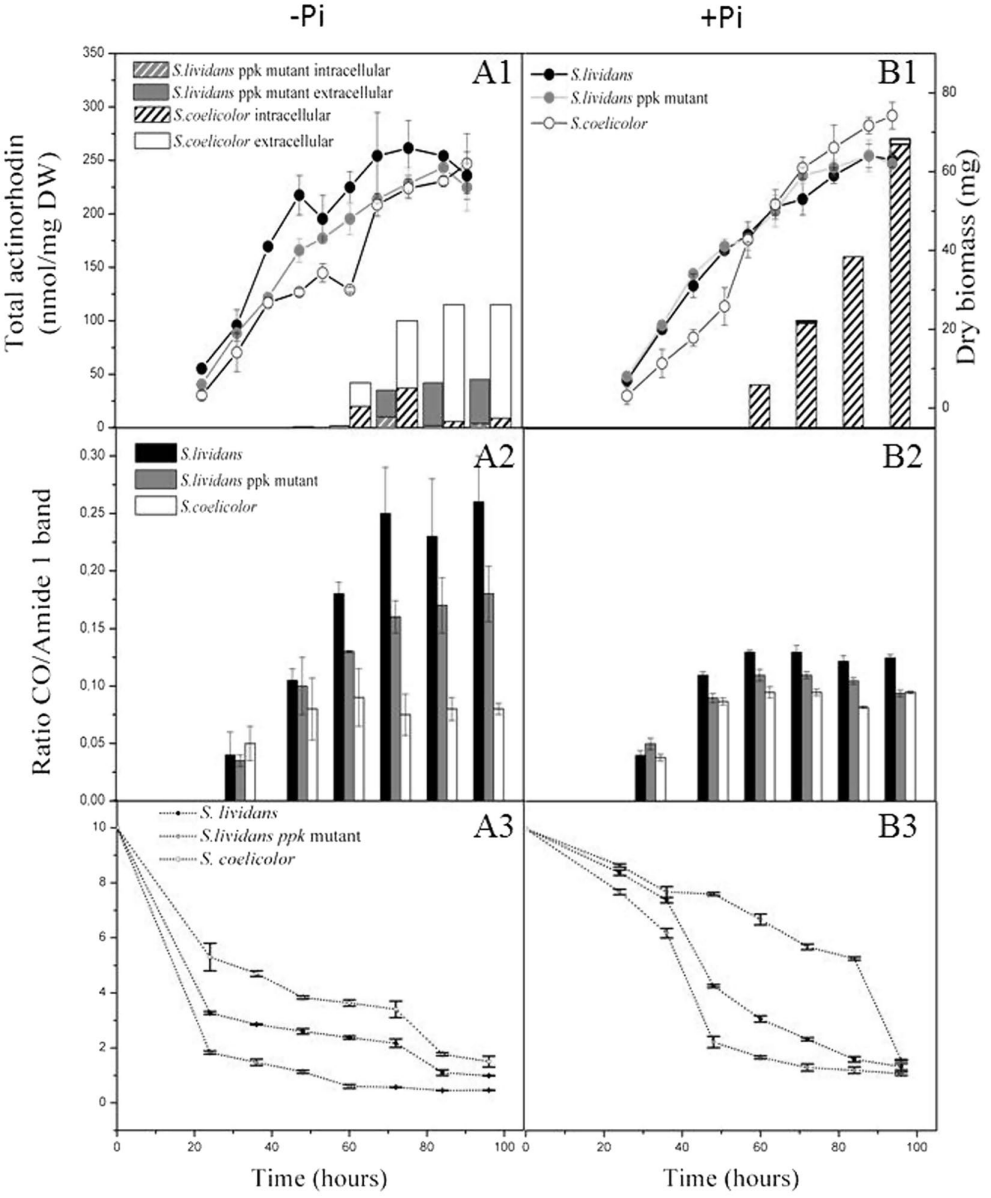
Cultures of the three strains were carried out in Pi limited (**A**) and Pi replete (**B**) conditions. (A1, B1) Estimation of growth (continuous lines, mg of dry biomass per plate) of *S. lividans* (black circles), the *ppk* mutant (grey circles) and *S. coelicolor* M145 (white circles), and evolution of the levels of ACT produced extracellularly and intracellularly by *S. coelicolor* (plain white and hatched histograms, respectively) and the *ppk* mutant of *S. lividans* (grey and grey hatched histograms, respectively) (A2,B2). Estimation of the evolution of the ratio of ester carbonyl band of lipids/amide I band of proteins representing the total lipid content per arbitrary unit of protein in *S. lividans* TK24 (black histograms), the *ppk* mutant (grey histograms) and *S. coelicolor* M145 (white histograms). (A3,B3) Estimation of the glucose concentration in the growth medium (continuous lines, mg of glucose per ml of medium) of *S. lividans* TK24 (black circles), the *ppk* mutant (grey circles) and *S. coelicolor* M145 (white circles).

Growth, total lipid and actinorhoddin production of *S. lividans* WT, its *ppk* mutant and *S. coelicolor* were followed throughout growth on Pi limited and Pi proficient R2YE solid medium (Fig. 1). The total lipid content of the lyophilized mycelia was quantified using Fournier Transformed Infra Red spectroscopy (FTIR) (see materials and methods). The total lipid content is mainly constituted by membrane lipids and storage lipids of the TriAcylGlycerol (TAG) family. Assuming that the content of membrane lipids does not vary greatly among the three strains, the difference in lipid content between strains likely reflects a difference in TAG content. The results showed that in Pi limitation, growth rate and biomass yield of *S. lividans* WT were slightly higher than those of the *ppk* mutant and *S. coelicolor* (Fig. 1A1). In *S. lividans*, TAG content steadily increased throughout growth, reaching a plateau at stationary phase (Fig. 1A2). TAG accumulation in the *ppk* mutant followed a similar temporal pattern but finally reached a 20% lower level of TAG than its parental strain (Fig. 1A2). In contrast, the lipid content of *S. coelicolor* remained rather constant and low throughout the cultivation period. From 72 h of cultivation, *S. coelicolor* had a total lipid content approximately 70% lower than that of *S. lividans* WT (Fig. 1A2). In these conditions, *S. coelicolor* and the *ppk* mutant, but not its WT parent, excreted the blue pigmented polyketide antibiotic, actinorhodin (ACT) at late growth stages. *S. coelicolor* produces on average 2 to 3 fold more ACT than the *ppk* mutant and this production started at least 12 h earlier (60 h versus 72 h) (Fig. 1A1) whereas its TAG content was at least 2 fold lower (Fig. 1A2).

In conditions of Pi proficiency, the strains had similar biomass yields than in Pi limitation (Fig. 1B1). The temporal pattern of total lipid accumulation was similar in the three strains, with a clear increase between 36 and 48 h and a rather constant content thereafter (Fig. 1B2). The overall TAG content of the two *S. lividans* strains was 1.5 to 2 fold lower than under Pi limitation whereas the TAG content of *S. coelicolor* was similar irrespective of Pi concentration (Fig. 1B2). The difference in TAG content between the three strains in Pi proficiency was still visible but was greatly reduced compared to conditions of Pi limitation (Fig. 1A2). This study thus indicated that, as found in other micro-organisms^26, 27^, Pi limitation is an important trigger for TAG accumulation in *S. lividans*. However, unexpectedly *S. coelicolor* escapes this quasi universal regulation.

In these conditions, the *S. lividans* strains did not produce any antibiotic while *S. coelicolor* produced large amounts of ACT. These remained mainly intracellular (Fig. 1B1) while being excreted in Pi limitation (Fig. 1A1). Previous studies showed that ACT remained exclusively intracellular when the pH of the medium was pH 4.5 to 5.5 but was excreted at pH values above 6^28, 29^. Consequently, the absence of ACT excretion is thought to be, at least in part, due to the pH of the medium, found to be acidic in Pi proficiency and neutral in Pi limitation (data not shown). In Pi proficiency, ACT production showed a continuous increase from 60 to 96 h, reaching a maximal production 2.5 fold higher than in Pi limitation. This surprising observation indicated that in *S. coelicolor*, ACT biosynthesis was not only resistant to repression by phosphate but was even stimulated by phosphate. In contrast ACT synthesis was highly sensitive to Pi repression in the *S. lividans ppk* mutant (Fig. 1B1) as in many, although not all^30^, other *Streptomyces* species^23–25^.

### *S. coelicolor* is characterized by an extremely high ATP/ADP ratio

**Figure 2:**
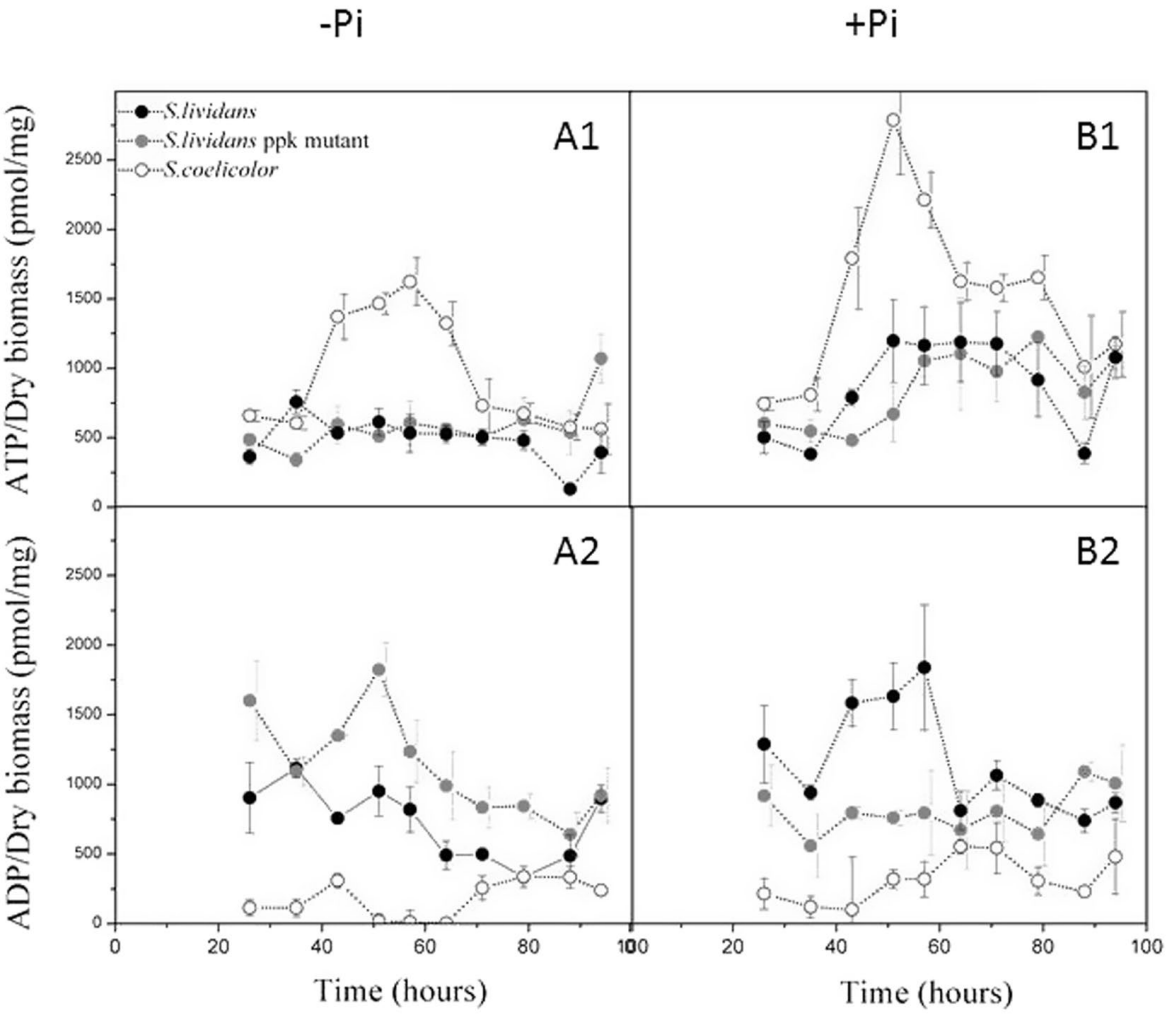
Quantification of intracellular ATP (A1,B1) and ADP (A2,B2) levels in *S. lividans* TK24 (black circles), its *ppk* mutant (grey circles) and *S. coelicolor* (open circles) grown in Pi limited (**A**) or Pi replete (**B**) conditions. The error bars represent the standard deviation of at least three independent experiments.

The ATP and ADP content of *S. lividans*, its *ppk* mutant as well as *S. coelicolor* was assayed at regular intervals during growth on a Pi limited and Pi replete medium (Fig. 2). In Pi limitation, the ADP content of the *ppk* mutant was higher than in the WT strain throughout growth (Fig. 2A2). This suggests reduced phosphorylation of ADP to ATP in this strain. This is consistent with Ppk acting as an ADPK in these conditions^14^. However, an ATP deficit that could have been expected was not observed in this strain. Indeed, the ATP content of the WT and *ppk* mutant of *S. lividans* was approximately the same up to 73 h of cultivation (Fig. 2A1). Interestingly, late in stationary phase (96 h) the *ppk* mutant showed a 2 fold higher ATP content than the WT strain (Fig. 2A1). This is thought to be due to re-oxidation of reduced co-factors generated by the β-oxidation of fatty acids resulting from TAG degradation^17^. Unexpectedly, the ATP content of *S. coelicolor* was 2 to 3 fold higher than in the *S. lividans* strains during active growth (between 43 and 65 h, Fig. 2A1), while the ADP content was extremely low throughout the cultivation period and hardly detectable at the time points where the intracellular ATP concentration was high (Fig. 2A2). This indicated an extremely efficient rate of conversion of ADP to ATP in *S. coelicolor*, likely due to high levels of oxidative phosphorylation.

In Pi proficiency, the overall ATP content of both *S. lividans* strains was rather similar and approximately 2 fold higher than in Pi limitation (Fig. 2B1). The ADP content of *S. lividans* WT was clearly higher than that of the *ppk* mutant up to 58 h of growth but then decreased to a level rather similar to that of the mutant, during continuous incubation (Fig. 2B2). This suggests that early in growth, the intracellular concentration of ATP being higher than in Pi limitation, the weakly expressed Ppk acts as a polyphosphate kinase (PPK) polymerizing the γ phosphate of ATP into polyphosphate and thus generating ADP^14^. In contrast, the overall ATP content of *S. coelicolor* was, as in Pi limitation, 2 to 3 fold higher than that of the *S. lividans* strains throughout growth but particularly between 40 h and 58 h (Fig. 2B1). The overall ADP content of *S. coelicolor* was much lower than in the *S. lividans* strains throughout the cultivation period (Fig. 2B2). *S. coelicolor* is thus unexpectedly characterized by an extremely high ATP/ADP ratio, observed both in Pi limitation and proficiency.

### The antibiotic producing strains consume their polyphosphate stores more actively than the non producing strain

**Figure 3:**
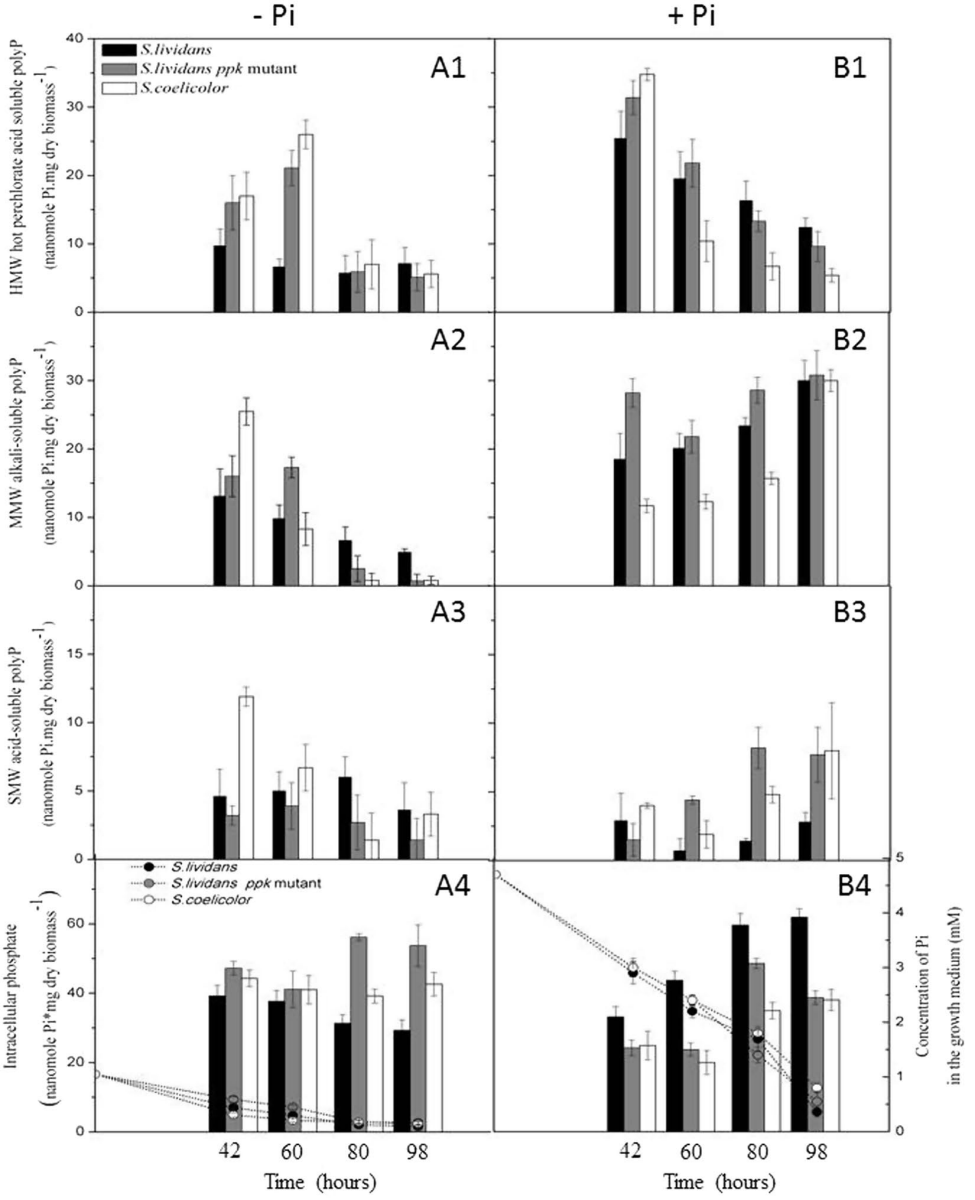
Cultures of the three strains on the surface of agar plates of R2YE medium in conditions of Pi limitation (**A**) or repletion (**B**). Phosphate and polyphosphate content of the original strain of *S. lividans*, its *ppk* mutant and *S. coelicolor* are represented by white, grey and black histograms, respectively. Polyphosphate was extracted with the Kulaev method that yielded three fractions whose evolution of Pi content was estimated (A1,B1) perchloric acid soluble fraction (HMW polyP); (A2,B2) alkali soluble fraction (MMW polyP); (A3,B3) acid soluble fraction (SMW polyP); (A4,B4) intracellular free phosphate and evolution of the concentration of Pi in the growth medium (dotted lines) of *S. lividans* (open circles), the *ppk* mutant (grey circles) and *S. coelicolor* (black circles).

Since active ATP synthesis requires the availability of free Pi, the kinetics of Pi uptake, as well as the intracellular content of free Pi and polyphosphate of was assayed in the three strains throughout growth on Pi replete and Pi limited R2YE medium (Fig. 3).

In Pi proficiency, the Pi uptake rates of the three strains, as deduced from the disappearance of Pi from the medium, were identical (Fig. 3B4). However, the dynamics of the levels of the different classes of polyP was different in the three strains throughout growth. High molecular weight polyP (HMW, i.e. hot perchloric acid soluble) is thought to be degraded into medium molecular weight polyP (MMW, alkali-soluble) itself then degraded into small molecular weight polyP (SMW, acid-soluble) and free Pi to fulfill metabolic needs. The content of HMW polyP, was high at 42 h in the three strains but then decreased more abruptly in *S. coelicolor* than in the *S. lividans* strains (Fig. 3B1). This clearly indicated a more active degradation of these Pi storage polymers in *S. coelicolor*. Similarly, the lower MMW polyP and the higher SMW polyP content in *S. coelicolor* (Fig. 3B2,B3) also indicated a more active degradation of these polymers. Interestingly, the *ppk* mutant had levels of HMW and MMW polyP similar to that of the WT strain but had clearly higher SMW polyP content than the latter. This suggested that in the wild type strain, SMW polyP is a substrate of Ppk, being used as Pi and energy donor to regenerate ATP from ADP (ADPK function). Indeed, in the absence of Ppk, SMW polyP degradation was shown to be slower in the *ppk* mutant than in the WT strain^15^. The lower intracellular content of free Pi in *S. coelicolor* and in the *ppk* mutant compared to WT *S.lividans* (Fig. 3B4) indicated a more active Pi consumption by these two strains.

In conditions of Pi limitation, the initial Pi uptake rate of the three strains was comparable (Fig. 3A4). The overall polyP content was lower in all strains than when Pi was replete. The HMW polyP content was higher in *S. coelicolor* and in the *ppk* mutant compared to the wild type *S. lividans* at 42 h and 60 h. This higher content might be linked to their active oxidative phosphorylation (see discussion). Indeed, several authors proposed that the synthesis of polyP, as that of ATP, directly involves oxidative phosphorylation^31, 32^. The content of MMW polyP of *S. coelicolor* and the *ppk* mutant decreased abruptly in the 42 h–60 h and 60 h–80 h periods, respectively (Fig. 3A2). The decay of SMW polyP followed a similar pattern in *S. coelicolor* and, to a lesser extent, in the *ppk* mutant (Fig. 3A3). Together these results demonstrated more active degradation of these Pi storage polymers in the two antibiotic producing strains compared to WT *S. lividans*.

In conclusion, our results indicated that, as anticipated, the overall polyP content of the strains was higher in Pi proficiency than in Pi limitation. *S. coelicolor* was shown to consume more actively its polyP stores than *S. lividans* WT, irrespective of Pi concentration and produced antibiotics in both conditions. The *ppk* mutant showed a pattern of polyP content and decay rather similar to *S. coelicolor* in Pi limitation where it produces antibiotics but more similar to *S. lividans* WT in Pi proficiency, where it does not produce antibiotics.

### *S. coelicolor* transports glucose less actively than the *S. lividans* strains

The kinetics of glucose uptake were determined throughout the cultivation period of the three strains during growth on Pi limited and Pi proficient R2YE media. Glucose uptake was shown to be more active in Pi limitation than in Pi proficiency for the three strains (Fig. 1A3,B3). In Pi limitation, the three strains took up glucose with similar kinetics starting by a phase of abrupt uptake, between 0 h and 24 h, followed by slower uptake phase, between 24 h and 76 h (Fig. 1A3). However, the *ppk* mutant took up glucose more actively than its parental strain that in turn imported glucose more actively than *S. coelicolor*.

In Pi repletion, the three strains maintained glucose uptake at a similar rate up to 36 h. An abrupt increase in the uptake of glucose took place in the two *S. lividans* strains between 36 and 48 h (Fig. 1B3) whereas in *S. coelicolor*, a similar abrupt uptake occurred only between 80 h and 96 h (Fig. 1B3). A high adenylate charge is known to inhibit glucose uptake and glycolysis in other organisms^33^. The lower glucose uptake in Pi proficiency than in Pi limitation, as well as the higher and lower glucose uptake of the *ppk* mutant and of *S. coelicolor*, respectively (Fig. 1A3,B3), are consistent with this regulation. The low glucose uptake of *S. coelicolor* likely results into a low glycolytic activity and thus insufficient levels of glycerol3P to promote TAG biosynthesis.

## Discussion

The aim of the present study was to define the metabolic features that are correlated with low, medium and high levels of biosynthesis of the blue pigmented polyketide antibiotic, actinorhodin (ACT), by phylogenetically closely related model *Streptomyces* strains. To do so, the similar and different features of carbon, phosphate and energetic metabolism of *S. lividans* TK24 WT, its *ppk* mutant and *S. coelicolor* M145, were determined.

In Pi limitation, *S. lividans*, a weak antibiotic producer, was characterized by a high TAG content. TAG being made from glycerol 3P and acetylCoA, originating from glycolysis, this indicated a predominantly glycolytic metabolism. In contrast *S. coelicolor*, a strong antibiotic producer, was characterized by a low TAG content and a high ATP/ADP ratio (Fig. 2). Consistently, this strain was shown to actively consume its polyphosphate stores to support high ATP generation indicating highly active oxidative metabolism (Fig. 3). Previous publications indeed reported that high antibiotic producing strains had a much lower polyP content than low producing strains^34–36^. The genetic causes of the highly active oxidative metabolism characterizing *S. coelicolor* remain to be elucidated. However, the phenotype of the *S. lividans ppk* mutant, which in conditions of Pi limitation, is similar to that of *S. coelicolor* with respect to polyP mobilisation, TAG content and antibiotic production, suggested the existence of some common regulatory features between these two strains.

The present study confirmed and extended *in vivo* our previous *in vitro* findings concerning the enzymatic function of Ppk^14^. Our results indicated that, when Pi is limiting, Ppk acts as an Adenosine Di Phosphate Kinase (ADPK), using polyphosphate as phosphate and energy donor to regenerate ATP from ADP. In the absence of this important ATP regenerating enzyme, ADP accumulates (Fig. 2A2) and the mutant strain likely experiences energetic stress. A reduced intracellular ATP concentration was therefore expected in the mutant however both *S. lividans* strains showed similar ATP levels throughout growth. This suggested that homeostatic processes, aimed at re-establishing the energetic balance, were triggered in the *ppk* mutant. We propose that the ATP shortage linked to the absence of Ppk induces a stimulation of glycolytic as well as oxidative metabolism, signaled by enhanced glucose uptake and consumption of polyP stores, respectively (Fig. 1A3–B3). In the *ppk* mutant, most of the pool of acetylCoA generated by glycolysis would be stored as TAG (Fig. 1A2) but a fraction of it would be used to fuel the Krebs cycle to restore the energetic balance of the cell. This process is accompanied by ACT biosynthesis as in *S. coelicolor*.

Notably, the production of ACT in the *ppk* mutant (Fig. 1A1,B1) was clearly repressed by Pi, as in most *Streptomyces* species^37^. In contrast, in *S. coelicolor*, ACT biosynthesis was not only resistant to repression by phosphate but was even stimulated by phosphate (Fig. 1A1,B1). However, both in Pi limitation or proficiency, when ACT production started, there was an immediate reduction in the levels of ATP. This temporal coincidence suggested that ACT biosynthesis is triggered in conditions of excessive oxidative phosphorylation activity and plays a role in down regulating the latter. ACT was shown to induce the expression of the small SoxR regulon that includes genes encoding NAD(P)H-dependent flavin or quinone reductases^38–40^. In an attempt to connect the induction of these proteins with the observed fall in ATP, we propose that, as reported in some other organisms^41, 42^, these proteins might be part of a redox loop that consumes (rather than generates) the proton motive force thus leading to a reduction of ATP generation. Alternatively, we cannot exclude that ACT, a benzochromanequinone-like molecule, like other molecules containing quinone groups (melanin or humic acid substances), could directly serve as alternative electron acceptors for the respiratory chain^43^ contributing to a less efficient functioning of the latter. In this respect, it is noteworthy that the calcium-dependent ionophore CDA^44^ as well as RED^45^, a red pigmented antibiotic of the prodigiosin family, other secondary metabolites produced by the *ppk* mutant^14^ and *S. coelicolor*, were also proposed to have energy dissipating function^46, 47^. Consequently, the deletion of the main antibiotic biosynthetic pathways in M1146, a derivative of *S. coelicolor* M145^48^, should result in higher ATP generation and thus better growth than the original strain. Indeed, our previous comparative fluxomic studies of M145 and M1146 carried out in minimal synthetic medium in bio-reactor demonstrated that M1146 yielded more biomass than M145^49^. This suggested that M1146 indeed generated more ATP than the original strain. Furthermore, the observed lower glucose uptake and thus lower flux through glycolysis of M1146 compared to M145^49^ is also consistent with the known inhibitory effects that high ATP levels exerts on glucose uptake and glycolysis^33^.

An important question is whether the well-defined *ppk* mutant can help us understand the related phenotype of *S. coelicolor*? The *ppk* mutant suffers energetic stress and activation of its metabolism takes place in order to re-establish the energetic balance. This activation is accompanied by reduced formation and/or mobilisation of carbon^17^ and polyP stores (this study) and by ACT as well as CDA and RED biosynthesis^14^. Since *S. coelicolor* is insensitive to the usual repression of antibiotic biosynthesis by phosphate, we propose that sensing/signaling of Pi availability and/or energetic state might somehow be altered in this strain. This strain would respond to a “presumed” energetic stress therefore triggering strong activation of oxidative metabolism to generate ATP.

On the basis of our results, we thus propose a novel view of the changes underlying the transition between primary and secondary metabolism in *Streptomyces*. In *Streptomyces* the switch from primary to secondary metabolism that is known to be triggered by energetic stress, would entail an activation of oxidative metabolism primarily aimed at the restoration of the energetic balance of the cell. To do so, the necessary fueling of the Krebs cycle by acetylCoA limits its availability for TAG accumulation and the re-oxidation of the reduced cofactors, generated by the Krebs cycle, by the respiratory chain requires the consumption of the polyphosphate stores to generate ATP. This results in low TAG and polyP content of the antibiotic producing strains. In this context, we propose that a function of ACT would be to tone down over-active oxidative phosphorylation to reduce ATP generation. This would contribute to growth slow down when some critical nutrients (especially phosphate) become scarce in the growth medium. This study thus raises the fascinating question that the secondary metabolites may have physiological functions for the producing strain other than competitive advantage. It is well established that the production of secondary metabolites accompanies growth slow down but our study suggests that the latter might play a role in this deceleration. This could be achieved through energy dissipation (this study) but perhaps also *via* other processes such as inhibition of DNA, RNA, protein and cell wall/membrane biosynthesis ﻿since. Numerous proteins involved in these processes constitute well known targets of various antibiotics^50^.

## Methods

### Bacterial strains, media and growth conditions

The strains used in this study were *S. lividans* TK24 WT^13^, *S. lividans* TK24 *ppk::hygro* strain^14^ and *S. coelicolor* M145^12^. 10^6^ viable spores of each strain were spread on the surface of 3 plates (42 mm diameter) of solid medium R2YE^51^ covered by a cellophane disk (Cannings Packaging Limited, Bristol, United Kingdom) and incubated at 28 °C. The medium was supplemented or not with K_2_HPO_4_. R2YE not supplemented with K_2_HPO_4_ contained 1 mM free Pi (Pi limitation) and that supplemented with K_2_HPO_4_ contains 5 mM Pi (Pi repletion), as determined with a PiBlue phosphate assay kit (Gentaur, France). At defined time points throughout growth, half of the wet biomass obtained from 3 plates was scrapped off the cellophane disks with a spatula and lyophilized. Growth was estimated as dry cell weight (DCW). The other half of each plate was used to extract and assay either polyP, ATP, ADP or actinorhodin.

### Determination of glucose, phosphate and actinorhodin concentration in the growth medium

To quantify glucose or phosphate uptake and excretion of actinorhodin in the surface grown cultures, the cellophane was lifted at defined time points and 3 agar cylinders of the growth medium were taken aseptically from each of the 3 replicates using an appropriate device. Samples were then incubated at 4 °C for at least 24 h in 1 mL of distilled water to allow diffusion of glucose, phosphate and actinorhodin into the aqueous phase. The concentration of glucose (glucose Assay kit from Sigma, ref GAHK-20) and phosphate (PiBlue phosphate assay kit from Gentaur) were determined according to the manufacturer recommendations. For extracellular actinorhodin assays, the supernatant was mixed with an equal volume of 2 M KOH and centrifuged. For intracellular actinorhodin assays, actinorhodin was extracted from the biomass upon incubation of the latter in 2 mL of 1 M KOH during 2 hours at 4 °C under agitation. This operation was renewed once. After centrifugation, the supernatant was mixed with HCl 4 M (1 M final concentration) leading to precipitation of actinorhodin. The red pellet was re-suspended in 1 M KOH. Extra and intra cellular concentration of actinorhodin was measured as A_640_ (e_640_ = 25320) and expressed as nanomoles of actinorhodin per mg of DCW^52^.

### Determination of total lipid content using ATR-FTIR measurements

To determine total lipid content, lyophilized mycelial samples of the *Streptomyces* strains were subjected to FTIR spectroscopy using a Bruker Vertex 70 FTIR spectrophotometer with a diamond ATR attachment (PIKE MIRacle crystal plate diamond ZnSe) and an MCT detector with a liquid nitrogen cooling system. A reference of 100 averaged scans was performed before each sample analysis. Scans were conducted from 3600 cm^−1^ to 600 cm^−1^ with a spectral resolution of 4 cm^−1^ with 100 averaged scans for each sample. This technique allows the establishment of spectral fingerprints of the complex biological structures under investigation. The C-H stretching bands of the CH_2_ of fatty acid chains (between 2959 and 2852 cm^−1^) and the C=O ester stretching band of the ester carbonyl (band near 1740 cm^−1^) are characteristic of lipids (neutral TAG as well as polar membrane lipids). The height of the sharp and distinct C=O ester stretching band of the ester carbonyl is especially relevant to monitor total intracellular lipid content of the strains^53^. Furthermore, since the biomass protein content can be directly characterized by the amplitude of the Amide I absorption band (1650 cm^−1^), all FTIR spectra can be normalized to this band allowing comparison of the total lipid content of mycelial lawns of different strains. The differences between the three strains for FTIR spectra, in terms of lipid content were clear and highly reproducible and are expressed as arbitrary units.

### Extraction and assay of intracellular ADP and ATP concentrations

In order to quantify intracellular concentrations of ATP and ADP, 50 mg of mycelium was collected in 2 ml screw-cap tubes (Sarstedt, 72.693.005), mixed with 1 mL of 5% cold perchloric acid, frozen in liquid nitrogen and stored at −70 °C. Glass beads (0.16 g, 0.5 mm diameter) were added to each tube and the mycelium was homogenized and broken by vigorous shaking (FastPrep®). Samples were kept at 4 °C, centrifuged at 16 000 g for 10 min. The potassium perchlorate precipitate was discarded and the supernatants were neutralized with KOH 34% and 3.4% and finally with potassium phosphate buffer 1 M at pH 7.5. The ATP and ADP concentrations ([ATP] and [ADP]) were determined by the luciferin-luciferase method using an ATP luminescence kit (CSLII, Roche) and a GLOMAX™ luminometer (model 2031-002, Turner BioSystems, Inc). Background [ATP] was measured by mixing 35 µL of Tris acetate buffer 330 mM (pH 7.75), EDTA 4 mM, K_2_SO_4_ 21 mM with 50 µL of luciferin-luciferase mix. The sample (10 µL) was added and after measuring luminescence, 10 µL of an internal ATP standard of *ad hoc* concentration (depending on the [ATP] in the sample) was added and luminescence measured again. [ATP] was determined directly according to a standard curve (0.1 to 10 µM) made in the same conditions. All assays were performed in triplicate in order to calculate standard deviation.

[ADP] was measured after enzymatic conversion to ATP with pyruvate kinase and phosphoenol pyruvate. The background of [ADP + ATP] was measured by mixing 35 µL of Tris acetate buffer 330 mM (pH 7.75), EDTA 4 mM, K_2_SO_4_ 21 mM, phosphoenol pyruvate 17 mM and pyruvate kinase 0.57 mg/mL (Roche) with 50 µL of luciferin-luciferase mix. The sample (10 µL) was added and after measuring luminescence, 10 µL of an internal ADP standard of *ad hoc* concentration (depending on the [ADP] in the sample) was added as above and luminescence measured again. [ADP + ATP] was determined according to an ADP (2 to 30 µM) standard curve made in the same conditions. [ADP] was calculated by subtracting [ATP] from [ADP + ATP]. Results were expressed as nanomoles of nucleotides per mg of DCW. All assays were performed in triplicate in order to calculate standard deviation.

### Extraction, fractionation and assay of Pi present in polyP fractions

The Kulaev method^54^ was used to extract polyP at defined time points throughout growth in Pi limited and Pi proficient conditions. This method yielded three fractions of polyP of different sizes. For this, 0.25 g of the harvested wet biomass was incubated in 5 ml of 0.5 N HClO_4_ at 4 °C for 30 min with continuous stirring, then centrifuged at 12,000 g for 15 min at 4 °C. Supernatant 1 contained the acid-soluble polyphosphate fraction (Small Molecular Weight polyP, SMW, approximately 10 residues long) as well as free Pi and nucleoside phosphate. The nucleoside phosphate was removed by absorption on activated charcoal Norit A from VWR. Pellet 1 was extracted with 5 ml of 0.05 N at 4 **°**C for 30 min and centrifuged in the same conditions as above yielding supernatant 2 and pellet 2. Supernatant 2 contained the alkali-soluble polyphosphate (Medium Molecular Weight polyP, MMW, 20 to 100 hundred residues long). The latter were precipitated by addition of barium acetate (pH 8.2) at a final concentration of 0.1 M, at 4 °C, for 24 h after adjusting the pH of the supernatant 2 to 8.2 with 0.1 N HCl. The suspension was centrifuged at 5,000 g for 30 min. The precipitate was dissolved in 1 ml of buffer (50 mM Tris-HCl at pH 8.0, 6 mM MgCl_2_) and solubilized using Dowex AG-50W (sodium form). Pellet 2 was treated with 5 ml of 0.5 N HClO_4_ for 30 min at 100 °C. This hot perchlorate extract contains acid- and alkali-insoluble polyphosphates, presumed to be High Molecular Weight polyP (HMW, several hundred residues long). The three fractions obtained were incubated with DNase I and RNase A, each at 350 µg.ml^−1^, in 5 mM MgCl_2_ for 3 h at 37 °C, then incubated with proteinase K, 750 µg.ml^−1^ at 37 °C for 2 h. The samples were subsequently extracted with phenol/chloroform (1:1, w/v equilibrated with Tris-HCl, pH 7.5) to remove proteins. The phases were separated by centrifugation at 14,000 g for 10 min. The aqueous phases (containing polyP) were extracted with chloroform, centrifuged in the same conditions, collected and stored at −20 °C. The amount of Pi and polyP present in the different fractions was determined by the estimation of the difference in the Pi content of the samples before and after complete hydrolysis of polyP upon incubation of the samples with 2 M HCl at 100 °C for 10 min and using the PiBlue phosphate assay kit. All assays were performed in triplicate in order to calculate standard deviation.

## References

[1] Bratkovič, N. R. a.T. (ed.) Future Antibiotic Agents: Turning to Nature for Inspiration. (ISBN 978-953-51-0723-1, InTech, 10.5772/33588, 2012).

[2] Watve MG, Tickoo R, Jog MM, Bhole BD (2001). How many antibiotics are produced by the genus Streptomyces?. Arch Microbiol.

[3] Chaudhary HS (2013). Antibacterial activity of actinomycetes isolated from different soil samples of Sheopur (A city of central India). J Adv Pharm Technol Res.

[4] Bachmann BO, Van Lanen SG, Baltz RH (2014). Microbial genome mining for accelerated natural products discovery: is a renaissance in the making?. J Ind Microbiol Biotechnol.

[5] Challis GL (2008). Mining microbial genomes for new natural products and biosynthetic pathways. Microbiology.

[6] Brotz-Oesterhelt H, Sass P (2010). Postgenomic strategies in antibacterial drug discovery. Future Microbiol.

[7] Duncan KR (2015). Molecular networking and pattern-based genome mining improves discovery of biosynthetic gene clusters and their products from Salinispora species. Chem Biol.

[8] Antoraz S, Santamaria RI, Diaz M, Sanz D, Rodriguez H (2015). Toward a new focus in antibiotic and drug discovery from the Streptomyces arsenal. Front Microbiol.

[9] Craney A, Ahmed S, Nodwell J (2013). Towards a new science of secondary metabolism. J Antibiot (Tokyo).

[10] Bai C (2015). Exploiting a precise design of universal synthetic modular regulatory elements to unlock the microbial natural products in Streptomyces. Proc Natl Acad Sci U S A.

[11] Chater KF (2016). Recent advances in understanding Streptomyces. F1000Res.

[12] Bentley SD (2002). Complete genome sequence of the model actinomycete *Streptomyces coelicolor* A3(2). Nature.

[13] Ruckert C (2015). Complete genome sequence of Streptomyces sp. CNQ-509, a prolific producer of meroterpenoid chemistry. J Biotechnol.

[14] Chouayekh H, Virolle MJ (2002). The polyphosphate kinase plays a negative role in the control of antibiotic production in Streptomyces lividans. Mol Microbiol.

[15] Ghorbel S (2006). Regulation of ppk expression and *in vivo* function of Ppk in Streptomyces lividans TK24. J Bacteriol.

[16] Ghorbel S, Kormanec J, Artus A, Virolle MJ (2006). Transcriptional studies and regulatory interactions between the phoR-phoP operon and the phoU, mtpA, and ppk genes of Streptomyces lividans TK24. J Bacteriol.

[17] Le Marechal P (2013). Comparative proteomic analysis of Streptomyces lividans Wild-Type and ppk mutant strains reveals the importance of storage lipids for antibiotic biosynthesis. Appl Environ Microbiol.

[18] Olukoshi ER, Packter NM (1994). Importance of stored triacylglycerols in Streptomyces: possible carbon source for antibiotics. Microbiology.

[19] Packter NM, Olukoshi ER (1995). Ultrastructural studies of neutral lipid localisation in Streptomyces. Arch Microbiol.

[20] Foley TL, Young BS, Burkart MD (2009). Phosphopantetheinyl transferase inhibition and secondary metabolism. FEBS J.

[21] Craney A, Ozimok C, Pimentel-Elardo SM, Capretta A, Nodwell JR (2012). Chemical perturbation of secondary metabolism demonstrates important links to primary metabolism. Chem Biol.

[22] Banchio C, Gramajo H (2002). A stationary-phase acyl-coenzyme A synthetase of *Streptomyces coelicolor* A3(2) is necessary for the normal onset of antibiotic production. Appl Environ Microbiol.

[23] Bibb MJ (2005). Regulation of secondary metabolism in streptomycetes. Curr Opin Microbiol.

[24] Liu G, Chater KF, Chandra G, Niu G, Tan H (2013). Molecular regulation of antibiotic biosynthesis in streptomyces. Microbiol Mol Biol Rev.

[25] Martin JF, Liras P (2012). Cascades and networks of regulatory genes that control antibiotic biosynthesis. Subcell Biochem.

[26] James AW, Nachiappan V (2014). Phosphate transporter mediated lipid accumulation in Saccharomyces cerevisiae under phosphate starvation conditions. Bioresour Technol.

[27] Valenzuela J (2013). Nutrient resupplementation arrests bio-oil accumulation in Phaeodactylum tricornutum. Appl Microbiol Biotechnol.

[28] Wright LF, Hopwood DA (1976). Actinorhodin is a chromosomally-determined antibiotic in Streptomyces coelicolar A3(2). J Gen Microbiol.

[29] Bystrykh LV (1996). Production of actinorhodin-related “blue pigments” by *Streptomyces coelicolor* A3(2). J Bacteriol.

[30] Curdova E, Jechova V, Zima J, Vanek Z (1989). The effect of inorganic phosphate on the production of avermectin in Streptomyces avermitilis. J Basic Microbiol.

[31] Pavlov E (2010). Inorganic polyphosphate and energy metabolism in mammalian cells. J Biol Chem.

[32] Tomaschevsky AA, Ryasanova LP, Kulakovskaya TV, Kulaev IS (2010). Inorganic polyphosphate in the yeast Saccharomyces cerevisiae with a mutation disturbing the function of vacuolar ATPase. Biochemistry (Mosc).

[33] Berg J. M, T. J., Stryer L. (eds) Biochemistry. 5th edition, http://www.ncbi.nlm.nih.gov/books/NBK22395/ (W H Freeman, New York; 2002).

[34] Kulaev IS, Bobyk AM, Tobek I, Goshtialek Z (1976). [The possible role of high molecular weight polyphosphates in chlortetracycline biosynthesis by Streptomyces aureofaciens]. Biokhimiia.

[35] Ziuzina ML, Kulaev IS, Bobyk MA, Efimova TP, Tereshin IM (1981). [Interrelationship of polyphosphate metabolism and levorin biosynthesis in Streptomyces levoris]. Biokhimiia.

[36] Telesnina GN, Krakhmaleva IN, Anisova LN, Bartoshevich Iu E, Sazykin Iu O (1986). [Valinomycin biosynthesis and the dynamics of the content of macroergic phosphorus compounds in Streptomyces cyaneofuscatus]. Antibiot Med Biotekhnol.

[37] Martin JF (2004). Phosphate control of the biosynthesis of antibiotics and other secondary metabolites is mediated by the PhoR-PhoP system: an unfinished story. J Bacteriol.

[38] Dela Cruz R (2010). Expression of the *Streptomyces coelicolor* SoxR regulon is intimately linked with actinorhodin production. J Bacteriol.

[39] Shin JH, Singh AK, Cheon DJ, Roe JH (2011). Activation of the SoxR regulon in *Streptomyces coelicolor* by the extracellular form of the pigmented antibiotic actinorhodin. J Bacteriol.

[40] Naseer N, Shapiro JA, Chander M (2014). RNA-Seq analysis reveals a six-gene SoxR regulon in *Streptomyces coelicolor*. PLoS One.

[41] Melo AM, Bandeiras TM, Teixeira M (2004). New insights into type II NAD(P)H:quinone oxidoreductases. Microbiol Mol Biol Rev.

[42] Simon J, van Spanning RJ, Richardson DJ (2008). The organisation of proton motive and non-proton motive redox loops in prokaryotic respiratory systems. Biochim Biophys Acta.

[43] Plonka PM, Grabacka M (2006). Melanin synthesis in microorganisms–biotechnological and medical aspects. Acta Biochim Pol.

[44] Lakey JH, Lea EJ, Rudd BA, Wright HM, Hopwood DA (1983). A new channel-forming antibiotic from *Streptomyces coelicolor* A3(2) which requires calcium for its activity. J Gen Microbiol.

[45] Malpartida F, Niemi J, Navarrete R, Hopwood DA (1990). Cloning and expression in a heterologous host of the complete set of genes for biosynthesis of the *Streptomyces coelicolor* antibiotic undecylprodigiosin. Gene.

[46] Haddix PL (2008). Kinetic analysis of growth rate, ATP, and pigmentation suggests an energy-spilling function for the pigment prodigiosin of Serratia marcescens. J Bacteriol.

[47] Danevcic T, Boric Vezjak M, Tabor M, Zorec M, Stopar D (2016). Prodigiosin Induces Autolysins in Actively Grown Bacillus subtilis Cells. Front Microbiol.

[48] Gomez-Escribano JP, Bibb MJ (2011). Engineering *Streptomyces coelicolor* for heterologous expression of secondary metabolite gene clusters. Microb Biotechnol.

[49] Coze F, Gilard F, Tcherkez G, Virolle MJ, Guyonvarch A (2013). Carbon-flux distribution within *Streptomyces coelicolor* metabolism: a comparison between the actinorhodin-producing strain M145 and its non-producing derivative M1146. PLoS One.

[50] Lewis K (2013). Platforms for antibiotic discovery. Nat Rev Drug Discov.

[51] Hopwood, D. A, B., M., Chater, K. F., Kieser, T., Bruton, C. J., Kieser, H. M., Lydiate, D. J., Smith, C. P., Ward, J. M. and Schrempf, H. Genetic Manipulation of Streptomyces: a laboratory Manual (Norwich: John Innes Foundation; 1985).

[52] Kieser, T., Bibb, M. J., Chater, K. and Hopwood, D. A. Practical Streptomyces genetics (Norwich, United Kingdom, 2000).

[53] Deniset-Besseau A, Prater CB, Virolle MJ, Dazzi A (2014). Monitoring TriAcylGlycerols Accumulation by Atomic Force Microscopy Based Infrared Spectroscopy in Streptomyces Species for Biodiesel Applications. J Phys Chem Lett.

[54] Vagabov VM, Trilisenko LV, Kulaev IS (2000). Dependence of inorganic polyphosphate chain length on the orthophosphate content in the culture medium of the yeast Saccharomyces cerevisiae. Biochemistry (Mosc).

